# Explicating peer feedback quality and its impact on feedback implementation in EFL writing

**DOI:** 10.3389/fpsyg.2023.1177094

**Published:** 2023-07-14

**Authors:** Wenjing He, Ying Gao

**Affiliations:** ^1^School of Foreign Languages, Northeast Normal University, Changchun, Jilin, China; ^2^Ministry of Education (MOE) Language Training Center and School of Foreign Languages, Northeast Normal University, Changchun, Jilin, China

**Keywords:** peer feedback quality, accuracy, revision potential, implementation, EFL writing

## Abstract

**Introduction:**

Although it is commonly acknowledged that peer feedback quality is crucial to the success of peer review, there is a lack of consensus on how it could be determined. More importantly, how feedback quality interacts with other factors like feedback features and focus, and ultimately influences peer feedback implementation remains insufficiently investigated.

**Methods:**

The present study examined peer feedback quality and its impact on Chinese students’ feedback implementation in two argumentative writing tasks. Peer feedback quality was measured according to a self-designed two-dimensional measurement scale: accuracy and revision potential.

**Results:**

Quantitative analyses of 5,606 implementable idea units of feedback and 440 writing drafts by 110 students revealed that feedback accuracy was at a medium level and revision potential was at a low level, with accuracy demonstrating stronger predictive power on implementation; the predictive strengths of feedback accuracy and revision potential were strongest when feedback features and focus were considered; the overall peer feedback quality was low and medium-quality feedback was implemented most frequently; feedback quality significantly and most strongly predicted implementation in combination with feedback features and focus.

**Discussion:**

The study highlights the importance of future instructions in training students to provide and implement high-quality feedback with good accuracy and high revision potential.

## Introduction

Despite the proliferation of studies on peer feedback over the past three decades (Tsui and Ng, 2000; Wu, 2019; Cui et al., 2021; Payant and Zuniga, 2022), doubts about the effectiveness of peer feedback remain constant “as students are not experts in a subject area, peer feedback is susceptible to variation” (Strijbos et al., 2010, p. 291). In particular, although the large class size in EFL contexts like China has necessitated the use of peer feedback as a complement to teacher feedback in writing courses (Hu and Zhang, 2014; Wu et al., 2022), this skepticism on feedback quality (Nilson, 2003; Gielen et al., 2010) has hindered the application of this instructional activity in such contexts.

The importance of peer feedback quality has been widely acknowledged (Hattie and Timperley, 2007; Walker, 2015; Rotsaert et al., 2018), but it has not been defined consistently in the literature (Rosalia and Llosa, 2009; Gielen et al., 2010; Hovardas et al., 2014). Generally, conceptualizations of feedback quality have shifted from a comment-centric perspective concentrating on the features, amount, and length of feedback (e.g., Sluijsmans et al., 2002; Prins et al., 2006; Patchan et al., 2018; Rotsaert et al., 2018) to a text-centric perspective which takes alignment and accuracy of peer feedback to text problems as central to feedback quality (e.g., van Steendam et al., 2010; Hovardas et al., 2014; Gao et al., 2019). More recently, feedback quality has been defined functionally by integrating problem detection accuracy with the usefulness of suggested solutions based on whether a comment could improve essay quality measurably on rubrics (Wu and Schunn, 2020a).

As success in peer review mostly relies on the use of feedback in subsequent revision/feedback implementation (van der Pol et al., 2008; Dressler et al., 2019), this integrative definition well highlights the significance of peer feedback in promoting the writing improvement. However, to capture more dimensions of peer feedback quality, a more detailed measurement is needed. Previous measurement scales from a comment-centric perspective generally focused on feedback types, features, and whether the feedback met certain assessment criteria (Prins et al., 2006; Walker, 2015), but they did not empirically test the contribution of the identified feedback characteristics on performance (Gielen et al., 2010). Thus, it is necessary to develop a comprehensive measurement scale that speaks specifically to the potential effect of peer feedback. Additionally, although feedback quality has been reported to determine implementation (van der Pol et al., 2008; Wu and Schunn, 2020a), it is still difficult to assume that this relationship would be similar in L2 writing classrooms, considering that language and culture may provide expected challenges (Carson and Nelson, 1994; Ramanathan and Atkinson, 1999; Lundstrom and Baker, 2009).

Therefore, in this study with Chinese EFL learners writing English argumentative essays, we intended to explicate peer feedback quality in measurable ways and test its impact on feedback implementation since using feedback to revise is central to peer review. Unlike most measurements concentrating on the characteristics of peer feedback (e.g., Sluijsmans et al., 2002; Prins et al., 2006), the current study focused on feedback content which can critically influence its effectiveness (Anson and Anson, 2017). We also considered two factors crucial to implementation: feedback features and focus, given that the revision process based on peer feedback is complex and feedback implementation is influenced by many factors (Wu and Schunn, 2020a). Specifically, we investigated whether considering these two factors would change the existing effect of feedback quality on feedback implementation.

## Literature review

### Peer feedback quality and its measurement

Although previous studies have shed light on peer feedback quality in the L1 context, understandings of feedback quality have been rather inconsistent (Rosalia and Llosa, 2009; Gielen et al., 2010; Wu and Schunn, 2020a). Generally, research on peer feedback quality mainly falls into three perspectives: comment-centric, text-centric, and integrative functional. Peer feedback quality from a comment-centric perspective was defined by the number and length of comments which could determine the sufficiency of persuasion (Patchan et al., 2018; Zong et al., 2020) or by the inclusion of helpful feedback features like identifying the problem, suggesting a solution, or explaining the problem (Sluijsmans et al., 2002; Prins et al., 2006; Li et al., 2010; Denton, 2018). Following this line of definition, Patchan et al. (2018) examined feedback quality by the amount of peer feedback using three different indicators: the number of words across comments provided, the overall number of comments, and the number of long comments. Along the same line, evaluating feedback quality with a measurement scale is the most commonly used measurement (Sluijsmans et al., 2002; Prins et al., 2006; Gielen et al., 2010). Generally, the measurement scales of feedback quality examine whether students’ feedback contains certain features helpful for students’ writing improvement. Frequencies or percentages of coded feedback features are usually compared within and across dimensions (Huang, 2018). However, it is problematic to define and measure feedback quality from a comment-centric perspective because there is no guarantee that the comments would orient toward text problems which mostly need to be solved in revision (Wu and Schunn, 2020a).

Peer feedback quality from a text-centric perspective focuses on the accuracy of comments in terms of both correctness and alignment. In the research by van Steendam et al. (2010), participants were asked to point out the problems in a text with 10–20 flaws and suggest revisions. The quality of feedback was determined by considering whether the problems were addressed in the correct ways in terms of “the correctness, exhaustiveness, and explicitness of student comments” (van Steendam et al., 2010, p. 321). Along the same line, Gao et al. (2019) examined the alignment of written peer feedback with text problems by coding each substance and high-prose text problem, and they found that the alignment between feedback and text problems significantly determined revision improvement. However, to exhaustively identify text problems is hard and often impossible, and the effect of accurate feedback can range from correcting the writing mechanics to substantially improving the essay content.

Hovardas et al. (2014) defined feedback quality by measuring both feedback accuracy and feedback features. This hybrid method combining the comment-centric perspective and the text-centric perspective sheds light on conceptualizing feedback quality from more than one dimension. Adopting an integrative functional approach, Wu and Schunn (2020a) defined feedback quality as the accuracy of problems detected and the usefulness of suggested solutions. This definition significantly highlighted the mediating role of peer feedback in guiding students to reflect on the original text and improve the revised text. Feedback quality was rated and categorized into high, medium, and low levels based on the likelihood that a particular comment would lead to essay improvement in measurable or non-measurable ways on a 7-point Likert scale. Wu and Schunn (2023) further investigated the effects of assessor writing performance on feedback quality by examining feedback problem identification accuracy and helpfulness of feedback. Though defining feedback quality from an integrative perspective has the strength of making holistic judgments, labeling feedback quality into high, medium, and low categories fails to reveal the finer grain sizes of feedback quality. For example, what are the specific criteria for determining accuracy levels? And how are the specific comment aspects leading to a meaningful improvement weighted in the rating?

Informed by the text-centric perspective and the integrative functional approach, this study proposed to define and measure peer feedback quality on two dimensions: accuracy and revision potential of peer feedback. The two-dimensional peer feedback quality speaks directly to the core of what teachers and students concern most: is the feedback accurate and has the potential to lead to writing improvement? Specifically, accuracy refers to both alignment of feedback to a text problem and its correctness in addressing the problem. Aligned and correct feedback is crucial for peers to improve their writing (Hovardas et al., 2014; Gao et al., 2019) because feedback that aligns with a text problem can be either correctly or wrongly given whereas if a seemingly correct/reasonable comment is not aligned with the text problem, it is useless for text improvement. Revision potential refers to the potential of peer feedback in leading to text improvement, which is explicated in detail by rating the extent to which peer feedback could lead to writing improvement at different levels because the revision potential of feedback may vary from improving a minor mechanic issue of writing to significantly improving the gist or the logic of the essay. Unlike Wu and Schunn’s (2020a) study which did not examine low-level writing issues (such as spelling and punctuation) due to a lack of statistical power, the present study investigated the feedback quality of the content issues and high-level writing issues (i.e., theme, text organization, and clarity of writing) as well as the low-level writing issues (i.e., grammar and mechanics) with different weights. In particular, content/high-level feedback was rated with higher revision potential in the measurement scale as it deserved more weighting in facilitating writing improvement. In argumentative writing, solid argumentation and reasoning are more challenging to students because the critical analysis of the facts and evidence imposes a heavy cognitive load on them (Noroozi, 2018; Latifi et al., 2021). Similarly, it may also be challenging to conduct a fair and objective assessment of complex content or high-level writing issues and comments may be limited to the surface-level issues without explanations for developing critical thinking (Noroozi et al., 2016; Latifi et al., 2021). Although peer feedback is guided by the review rubric and related to the original text, the choice of feedback focus on simple or complicated issues is made by the students.

### Impact of peer feedback quality on implementation

Peer feedback implementation refers to students’ incorporation of peer feedback in revising their written text (Dressler et al., 2019), which is the linchpin of peer review. However, there are still uncertainties over whether or why students implement peer feedback in revisions (van der Pol et al., 2008; Walker, 2015; Wu and Schunn, 2020b). Generally, student writers are more likely to use more elaborated feedback (Noroozi et al., 2016), feedback with concrete suggestions (van der Pol et al., 2008), feedback which aligns with the text problems (Gao et al., 2019) and feedback helpful to writing improvement (Wu and Schunn, 2020a).

The effectiveness of peer feedback in terms of successful implementation hinges at least partly on the quality of the feedback that students provide (van der Pol et al., 2008, p. 1805). Hovardas et al. (2014) reported that students selectively used accurate feedback because they validated the effectiveness of feedback by cross-checking peer feedback and teacher feedback. Gao et al. (2019) found that whether the feedback aligned with the actual text problem or not could pose an impact on students’ revision improvement as the revision was found to be consistent with the feedback received. By judging whether the peer feedback had enough potential to generate a meaningful improvement in the text being reviewed, Wu and Schunn (2020a) found that students were more likely to implement feedback when both feedback quality and frequency increased.

Studies have shown that feedback quality is essential to students’ use of feedback, but the size of the effect is not clear and the specific contributions of accuracy and revision potential remain unexplored. Practically, with the increasing use of peer feedback among Chinese student writers (e.g., Gao et al., 2019; Li and Zhang, 2021), it is crucial to comprehend how feedback quality influences Chinese students’ feedback implementation in order to improve the suggestions offered to students on how to provide constructive feedback. Additionally, to comprehensively explicate feedback quality and its impact on implementation, we also investigated other variables that may contribute to the dynamics and variation of the impact of feedback quality on feedback implementation, namely, feedback features, focus, gender, and comment length.

### Peer feedback features

In addition to feedback quality, feedback implementation could be influenced by other factors such as students’ perceptions (van der Pol et al., 2008; Kaufman and Schunn, 2011), feedback focus (Shi, 2021), and individual differences (Winstone et al., 2017). One of the most important factors influencing feedback implementation is feedback features which refer to the structural components of feedback, such as whether they explicitly describe a problem or give praise (Wu and Schunn, 2020b). A large number of categorization systems have been utilized to investigate feedback features (e.g., Nelson and Schunn, 2009; Gielen and de Wever, 2015; Elizondo-Garcia et al., 2019). Psychologically, feedback features can be both cognitive (i.e., summarization, suggestion, explanation) and affective in nature (i.e., praise, mitigating praise) (Nelson and Schunn, 2009).

The impact of feedback features has been reported to be rather complicated. Some implementable features targeting the text problems (i.e., identification of the problem, solutions to address the problem) can be helpful to peers as they can arouse thinking, reflections, critical thinking (Filius et al., 2018), and implementation. Identification of problem (Lu and Law, 2012), suggestion (Nelson and Schunn, 2009; Leijen, 2017), solution (Wu and Schunn, 2020b), and explanation (Gielen et al., 2010; Wu and Schunn, 2020a) have been reported to pose a positive effect on feedback implementation in some studies. By contrast, other studies have reported that there is a negative impact of solution (Patchan et al., 2016) and explanation (Tseng and Tsai, 2007; Nelson and Schunn, 2009) on feedback implementation. However, peer feedback quality has not been considered when determining which feedback features are crucial to feedback implementation (Wu and Schunn, 2020a), which might be one explanation for the inconsistent earlier findings. Possibly, the effect of peer feedback is determined by feedback quality in the first place as inaccurate feedback might not be used no matter how many useful features it contains. Conversely, it is also possible that containing more helpful features (i.e., explanation of the problem) would increase the possibility of implementation even if the feedback does not fully address the text problem. For example, praise in a critical comment may persuade a peer to act upon it even if it is inaccurate (Wu and Schunn, 2020a).

Although feedback features are not the central focus of the current study, feedback features must also be carefully controlled because how peer feedback is structured would influence students’ judgment about its persuasiveness and usefulness. Therefore, this study attempts to extend the current knowledge of feedback quality by considering feedback features when examining what contributes to feedback implementation.

### Peer feedback focus

Another important variable that especially relates to feedback implementation is peer feedback focus. It refers to the topic of the issue described in feedback such as grammar, thesis, and sufficiency of the examples (Patchan et al., 2016). Broadly, peer feedback can be divided into content focus and writing focus (Patchan et al., 2016; Gao et al., 2019). The content focus of feedback is concerned with meaning issues such as missing content, whereas the writing focus involves both high-level and low-level writing issues such as clarity and transitions of the ideas (Patchan et al., 2016; Gao et al., 2019). Content and high-level feedback focuses on aspects like argumentation, flow, and organization whereas low-level feedback covers aspects like mechanics, formatting, tense, and plurals (Allen and Mills, 2014).

Feedback focusing on meaning/content, or high-level and low-level writing issues varies both in cognitive load and feedback implementation rate of feedback, as well as in the effect to improve revision quality (e.g., Baker, 2016; Patchan et al., 2016). Patchan et al. (2016) reported that a writer tended to improve revision quality by implementing high-level feedback. Although implementing high-level feedback is more beneficial to learning cognitively, it usually requires more learner effort (Ene and Upton, 2014; Baker, 2016). Additionally, learners tend to implement more form focus or low-level feedback and less high-level feedback (e.g., Tsui and Ng, 2000; Allen and Mills, 2014; Gao et al., 2019). Gao et al. (2019) found that students repaired a larger number of less challenging problems while ignoring the more demanding content and high-level writing problems, indicating that complex content feedback or high-level feedback sometimes might be beyond learner means.

Feedback focus may have an impact on the relationship between feedback implementation and feedback quality. High-quality feedback that may lead to a meaningful text improvement might not be implemented if it requires major revision on the writing content or the overall writing organization and logic because the revision is cognitively demanding and requires more learner effort. Thus, when investigating the impact of feedback quality on students’ implementation, feedback focus should be considered.

### Additional variables

In addition to feedback features and focus, other variables like gender, comment length, and first draft quality may also influence feedback implementation and therefore should be statistically controlled (Noroozi et al., 2020; Wu and Schunn, 2020b). Gender has been found relevant to peer review as students of different gender might respond to peer feedback differently (Prinsen et al., 2009; Noroozi et al., 2020; Wu and Schunn, 2020b; Wu and Schunn, 2021). Noroozi et al. (2020) found that gender could influence feedback quality, essay quality, and students’ learning of writing content. Prinsen et al. (2009) found that males disagreed with their learning partners more frequently than females and males expanded on their messages less than women.

Comment length might influence student writers’ perceived feedback quality and thus influence feedback implementation (Patchan and Schunn, 2015; Patchan et al., 2018). Students are more likely to reflect on the long and detailed feedback received and perceive a stronger need to make any revisions (Zong et al., 2020).

First draft quality may influence the feedback amount, type, and the likelihood of implementation (Hovardas et al., 2014; Patchan et al., 2016; Wu and Schunn, 2023). For instance, the author may receive less implementable feedback simply because the draft is of good quality and has fewer text problems. Thus, when examining the effect of feedback quality, it is essential to control the first draft quality.

Although much is now known about the influencing factors of feedback implementation, less is known about the role of feedback quality. More importantly, there is not enough work that combines the two dimensions of accuracy and revision potential in explicating feedback quality and its effect on implementation. Further, even less is known about whether, and if so how, the effect of feedback quality changes when other interacting factors are considered.

## Research questions

The current study examined the impact of peer feedback quality on feedback implementation by taking both feedback features and focus into consideration. Specifically, the following two research questions were addressed:

1. What is the relative contribution of feedback accuracy and revision potential to feedback implementation with the consideration of feedback features and focus?
2. What is the relationship between the two-dimensional feedback quality and implementation with the consideration of feedback features and focus?

## Methods

### Participants and settings

This study was conducted in a compulsory course called “Comprehensive English” at a research-intensive university in Northeast China. The course was offered at Fall semesters to first-year graduate students majoring in computer science and communication once a week for three class periods. The course aimed to cultivate students’ comprehensive language skills, with a particular focus on reading and writing. An asynchronous online peer review platform (*Peerceptiv*) was used in organizing writing peer review activities. *Peerceptiv*^1^ is a research-validated and data-driven peer learning tool to assist students in demonstrating disciplinary knowledge through writing feedback practices (Li, 2023). It was developed over a decade of peer learning research at the Learning Research & Development Center at University of Pittsburgh. It is used to implement peer learning in North America and around the world in the sciences, English language arts, business and almost every other subject matter. To guarantee objective review and active engagement of the students, the drafts were randomly and anonymously distributed among peers in a double-blinded manner.

The 116 students were a convenience sample of enrollees in the course in two intact classes. Six students were excluded because they failed to submit drafts or review peers’ essays, leaving 110 in the study (60 in Class A and 50 in Class B). Students’ age ranged from 21 to 29 (*M* = 23.65). All the students passed the national English graduate record examination (NEGRE, with a possible total of 100 points) (*M* = 65.13, *SD* = 6.40). In general, the L2 proficiency of the students was approximately between 72 and 100 on the Test of English as a Foreign Language (TOEFL), which corresponds to the intermediate level. Results from the independent samples *t*-test revealed that students in the two classes had no significant difference in English proficiency based on their test scores in NEGRE (Class A: *M* = 65.74, *SD *= 6.34; Class B: *M* = 64.50, *SD* = 7.84) (*t* = −1.84, *df* = 73.09, *p* > 0.05). All students were taught by the same teacher and they all agreed that their data could be used for research.

### Procedures

#### Training procedures

Peer review training is important for students to define clear objectives and remove misconceptions about the reviewing rubric. Consequently, peer review training activities were carried out to assist students to understand the processes of peer review, get familiarized with *Peerceptiv*, and motivate students to engage in peer review.

Students were trained as a group in class. Training procedures consisted of four steps: watching a short video introduction to *Peerceptiv*; teacher modeling through analyzing sample essays and components of high-quality feedback; teacher lectures on the benefits and ways of being a good reviewer and teacher-guided discussion on implementing feedback to improve writing. Additionally, consistent help was provided after class to help students with difficulties in the reviewing process. Supplementary Appendix B summarizes the training steps.

#### Writing and reviewing procedures

Participants completed three main tasks. They submitted the first draft to the *Peerceptiv* platform, then provided feedback for three peers’ essays, and finally revised their own draft based on peer feedback. Writing and reviewing activities on two writing tasks were conducted in this study. The two writing topics were: (1) “Some working parents believe childcare centers can provide the best care for their children, others believe that family members like grandparents can do a better job. Which do you prefer?” (Week 3); (2) “Do you agree or disagree with the following statement? One should never judge a person by external appearance” (Week 8). For each topic, students were asked to write a five-paragraph argumentative essay in 250–300 words in English. The essay was expected to include an introduction of the topic, solid evidence and examples, possible counterarguments and rebuttals, and a concise conclusion.

Writing and reviewing activity for each writing task lasted for 4 weeks. After writing and submitting draft one to *Peerceptiv* in the first week, students were given 2 weeks to read and review three peers’ texts in English based on a four-dimension reviewing rubric which includes the thesis statement, organization, argument, and grammar and vocabulary (Supplementary Appendix A). The reviewing rubric was developed and adapted by following the previous reviewing rubric in Gao et al. (2019), Wu and Schunn (2020a), and Li and Zhang (2021). A minimum of three comments was required in each dimension. In the fourth week, students revised their own drafts before submitting the revised draft to the platform. Consequently, each student completed 4 writing drafts (2 for each topic) and 2 rounds of peer review (1 for each topic) in an 18-week semester.

### Measures

#### Feedback coding

To precisely examine feedback quality, implementation, and other variables, all feedback comments were first segmented into idea units because a reviewer may provide several revision ideas in a single dimension (Wu and Schunn, 2021). An independent idea unit was defined as raising and/or solving one problem on one dimension (Wu and Schunn, 2020b). The comments were segmented by two research assistants who discussed with the authors the precision of segmentation constantly and solved all the disagreements. In total, the comments were divided into 8,107 idea units, among which 5,606 were implementable feedback. Implementable feedback could lead to revisions while non-implementable feedback could not (i.e., feedback including only praise). Since this study focused on feedback implementation, only implementable feedback comments were further analyzed and therefore praise and summary were excluded. The same two research assistants double-coded all the implementable feedback by following the rating and coding schemes (Tables 1, 2), and disagreement was resolved through discussions together with the authors. *Kappa* values for each of the coding categories ranged from 0.70 to 0.90, indicating high inter-rater reliability.

**Table 1:** Measurement scale of peer feedback quality.

Dimension	Score	Description	Example
Accuracy (*K* = 0.75)	0	Feedback that is not aligned with the text problem	“In this day and age, childcare centers are becoming more and more professional,” “professional” should be in noun form.
	1	Feedback that is aligned with the text problem but incorrectly addresses it	In the second sentence, “There is a discussion about whether children should be sent to childcare center or be looked by their grandparents at home.” “looked by” should be “looked at by.”
	2	Feedback that is aligned with the text problem but only correctly addresses part of it	There aren’t topic sentences in the three body paragraphs. I think you should add “The childcare center can enhance children’s communication ability” in the beginning of the second paragraph. But I do not know how to revise your third and fourth paragraph.
	3	Feedback that is aligned with the text problem and correctly addresses it	You may add some counter-arguments and rebuttals to support your position, which means, instead of talking about the benefits childcare centers have, you can list some defects when grandparents take care of children.
Revision potential (*K* = 0.70)	0	Feedback that has no potential of leading to any writing improvement or has the potential of leading to negative changes	The word “traveled” should be changed to “travelled.”
	1	Feedback that has the potential of leading to minor writing improvement through solving a singular low-level writing problem	I think in the first paragraph, the word “today” should be capitalized.
	2	Feedback that has the potential of leading to writing improvement through solving a common low-level problem or a singular content/high-level writing problem	The writer did not make full explanation of the examples in the second paragraph because he failed to give the reasons why childcare centers make kids more independent than peers. He could write that childcare centers could train kids to get dressed by themselves.
	3	Feedback that has the potential of leading to significant improvement of writing through solving a holistic content or high-level writing issue	This article lacks two paragraphs. The second paragraph should be divided into three paragraphs. You can talk about the professionalism of childcare centers in para 2, how childcare centers help children develop their abilities in para 3 and add some counter-arguments in para 4.

**Table 2:** Coding scheme of feedback features, focus, and implementation.

	Definition	Examples
Feedback features		
Identification (*K* = 0.90)	Feedback identifying a text problem	The first paragraph is too long.
Suggestion (*K* = 0.83)	Feedback giving general advice for revision	You should pay attention to the punctuation.
Solution (*K* = 0.79)	Feedback providing a specific solution for revision	In the second para, “,” should be changed to “.”
Explanation (*K* = 0.82)	Feedback containing an explanation of an issue	The word “external” in the first paragraph can be removed because the word appearance itself has the meaning of external.
Mitigating praise (*K* = 0.71)	Feedback on a text problem containing a praise	It is great to associate this topic with the mental health of teenagers and value formation. But the argument process still needs to be strengthened.
Feedback focus		
Meaning-level/surface-level (*K* = 0.89)	Feedback on thesis, evidence, argument, organization, or conclusion/Feedback on convention, grammar, sentence variety, word choice, cohesion, and reference	M: The conclusion is a little short. S: In the first para, “matters” should be changed to “matter.”
Implementation (*K* = 0.77)		
Implemented	Feedback that is incorporated in the revision	Add a title. (The author added a title in draft 2).
Not implemented	Feedback that is not incorporated in the revision	There is no thesis statement. (The author did not add the thesis statement in draft 2).

#### Feedback quality

Based on our proposed definition, a two-dimensional measurement scale was developed (Table 1). Each idea unit was checked to see whether it aligned with the text problem, whether it correctly addressed the problem, and whether it had the potential to lead to text improvement.

To quantify feedback quality, both the accuracy and the revision potential of feedback were rated on a 0–3 scale, each with a description and an example in Table 1. The best feedback which accurately addressed a problem and could lead to significant improvement of writing through solving a holistic content or high-level writing issue would get 6 points in rating while the worst feedback would get 0 points.

For instance, the feedback “*The writer did not make full explanation of the examples in the second paragraph because he failed to give the reasons why childcare centers make kids more independent than peers. He could write that childcare centers could train kids to get dressed by themselves.*” was rated as 5-point quality feedback as in accuracy it got 3 points for accurately addressing the problem and 2 points in revision potential for leading to writing improvement by solving a singular content problem. In another idea unit “*In the second sentence, ‘There is a discussion about whether children should be sent to childcare center or be looked by their grandparents at home’. ‘looked by’ should be ‘looked at by’*.” was only assigned 1 points. The idea unit aligned with a grammatical error in the essay (word collocation of “*look*”), but it incorrectly addressed the text problem (The correct form should be “*looked after by*”). Therefore, the idea unit only got 1 points in accuracy and 0 points in revision potential since the sentence was still wrong if the feedback was implemented.

#### Feedback features

Feedback was coded for the presence/absence of five feedback features, namely, identification, suggestion, solution, explanation, and mitigating praise (Table 2 for definitions and examples). We coded “1” for the presence and “0” for the absence.

#### Feedback focus

Feedback was coded as meaning-level (content and high-level) feedback if it focused on the thesis, argument, evidence for claims, conclusion, and organization. Feedback on word choice, grammar, cohesion, sentence variety, and conventions was labeled surface-level (low-level) feedback. Since each feedback either focused on meaning-level or surface-level issues, it was binary-coded, “1” for meaning-level and “0” for surface-level.

#### Feedback implementation

Feedback implementation was coded for whether the feedback was implemented in the revised drafts. The changes between the first and the revised draft were located using MS Word’s Compare Document function. If a text change was made in response to the feedback, the feedback was coded as implemented. The feedback was labeled “Not Implemented” if it did not seem to lead to any revisions.

#### Text quality

Students’ first draft writings were rated and calculated by the mean value of ratings from the same two assistants who coded the feedback. Following ESL Composition Profile (Jacobs et al., 1981), the scoring rubric covered content, organization, vocabulary, language use, and mechanics which were in good alignment with the review prompt questions provided to students. The *Kappa* values of the two raters for the first drafts in the two tasks were 0.77 and 0.82, respectively.

#### Comment length

Comment length refers to the number of words in each piece of feedback (Patchan and Schunn, 2015), calculated by the function of MS Excel automatically. The average feedback length was 18.16 words.

### Data collection and analysis

The writing drafts and peer feedback were downloaded from *Peerceptiv*. In total, we examined 440 writing drafts from 110 students in two tasks and 5,606 implementable feedback. Variables and their descriptions were summarized in Table 3.

**Table 3:** Types of coding and measures of variables in the study.

Variable	Type	Description
Dependent variable		
Implementation	Binary	Whether the feedback is used in revisions or not
Independent variables		
Feedback quality		
Accuracy	Continuous	Whether the feedback accurately addresses the text problem
Revision potential	Continuous	To what extent the feedback could lead to writing improvement
Feedback features		
Identification	Binary	Whether the feedback identifies the text problem or not
Suggestion	Binary	Whether the feedback provides general advice for revision or not
Solution	Binary	Whether the feedback provides a specific solution for revision or not
Explanation	Binary	Whether the feedback contains an explanation or not
Mitigating praise	Binary	Whether the feedback on a text problem includes praise or not
Feedback focus		
Meaning-level/surface-level	Binary	Whether the feedback is about meaning-level issues or surface-level issues
Control variables		
Gender	Binary	Whether the student is female or not
Comment length	Continuous	Number of words in an idea unit
Draft 1 quality	Continuous	Mean ratings across two writing experts

To address the first research question, a basic description of data such as peer feedback quality, features, focus, and implementation was presented (Table 4) and SPSS 26.0 was used to conduct statistical analysis. Since the feedback data (i.e., features, quality) was nested within authors, two-level hierarchical modeling was conducted with Stata 15. Logistic regression was used because the dependent variable (peer feedback implementation) was a binary outcome variable. The first set of regression was conducted to analyze how the two dimensions of feedback quality predicted feedback implementation. To answer our second research question, the second group of logistic regression was conducted to explore how the overall peer feedback quality predicted feedback implementation.

**Table 4:** Means and standard deviations of feedback quality, features, focus, and implementation.

Measure	M	SD	Min	Max
Peer feedback quality	3.36	1.93	2.36	4.32
Accuracy (0–3)	2.07	1.24	1.36	2.78
Revision potential (0–3)	1.29	0.94	0.91	2.01
Peer feedback features				
Identification	26.46	11.21	5	55
Suggestion	14.49	5.40	5	28
Solution	19.80	7.79	6	43
Explanation	1.56	1.76	0	11
Mitigating praise	0.53	0.85	0	4
Peer feedback focus				
Meaning-level	23.91	8.20	6	41
Surface-level	27.05	9.00	8	49
Peer feedback implementation	23.94	9.29	4	46

Since logistic regression was used, the results of the models were presented as odds ratios (*OR*). An odds ratio (*OR*) is a measure of the association between an exposure and an outcome. The exponential function of the regression coefficient is the odds ratio associated with a one-unit increase in exposure. Feedback features and focus were also considered in both sets of regressions to test the interactive strength of prediction on feedback implementation.

## Results

In this section, we first reported the levels of accuracy and revision potential of feedback, as well as the descriptive data of feedback features, focus, implementation and other control variables. We then reported the correlations among different variables and finally reported the relative contribution of feedback accuracy and revision potential to feedback implementation. We reported the findings of the second research question by following similar procedures.

### Relative contribution of feedback accuracy and revision potential to feedback implementation

According to the two-dimensional measurement scale, feedback quality was measured on both accuracy and revision potential of feedback toward text problems. It was found that average feedback accuracy (*M* = 2.07, *SD* = 1.24) was at a medium level (approaching 70% of the total rating). Specifically, 62.5% of the feedback (*N* = 3,505) aligned with and accurately addressed the text problems (rated as 3), and 20.0% (*N* = 1,041) of the feedback was not aligned with text problems (rated as 0). Revision potential of feedback (*M* = 1.29, *SD* = 0.94) was at a low level (getting about 41% of the total rating). Only 12.5% (*N* = 705, rated as 3) had the potential for significant improvement in writing, and 21.7% of feedback (*N* = 1,221, rated as 0) would not lead to text improvement. In particular, for feedback with 3 points in accuracy (*N* = 3,505), only 16% (*N* = 577) got 3 points and about 50% (*N* = 1,910) got 1 point in revision potential. This big inconsistency between accuracy and revision potential suggests that accurate feedback may not lead to big text improvement due to limited revision potential.

Table 4 presents a summary of the descriptive data averagely on each author. With feedback quality, we reported the average rating. With feedback features, focus, and feedback implementation, the average amount of feedback by the authors was reported.

Among the feedback features, identification (*M* = 26.46) and solution (*M* = 19.80) were the most common, while mitigating praise was the least frequent (*M* = 0.53). Moreover, students received significantly less meaning-level feedback than surface-level feedback according to paired samples *t*-test (*t* = −4.47, *df* = 109, *p* < 0.01). Of the 5,606 implementable feedback analyzed, 2,633 (47%) was implemented. Each author averagely incorporated 23.94 feedback.

Before running the regression tests, a correlation analysis was conducted among the variables (Table 5). Both accuracy and revision potential were significantly related to feedback implementation (*r*_accuracy_ = 0.38**; *r*_revision potential_ = 0.21**). Additionally, suggestion (*r* = −0.08**), solution (*r* = 0.11**), feedback focus (*r* = −0.11**), and first draft quality (*r* = −0.02*) significantly correlated with implementation.

**Table 5:** Correlations among two dimensions of feedback quality (accuracy and revision potential), features, focus, and implementation.

	Variable	1	2	3	4	5	6	7	8	9	10	11
1	Accuracy											
2	Revision potential	0.56**										
3	Identification	−0.15**	0.16**									
4	Suggestion	0.24**	0.30**	−0.21**								
5	Solution	0.09**	−0.41**	−0.45**	−0.49**							
6	Explanation	0.08**	0.03**	−0.04**	−0.01	−0.01						
7	Mitigating praise	0.01	0.06**	−0.02	0.07**	−0.06**	0.01					
8	Meaning-level (reference: surface-level)	0.02	0.41**	0.29**	0.32**	−0.56**	0.04**	0.07**				
9	Gender	0.00	−0.03**	0.02	−0.01	0.01	−0.03**	−0.02	−0.02			
10	Comment length	0.11**	0.02	0.08**	0.13**	0.07**	0.22**	0.09**	0.15**	−0.03*		
11	Draft 1 quality	−0.05**	−0.09**	−0.02	0.02	0.01	−0.01	−0.02	−0.02*	0.20**	−0.04**	
12	Implementation	0.38**	0.21**	−0.01	−0.08**	0.11**	0.02	−0.01	−0.11**	0.02	−0.01	−0.02*

*p < 0.05, **p < 0.01.

To further explore the predictive strength of peer feedback accuracy and revision potential as well as the other variables, the first set of logistic regression test was run (Table 6). Model 1 included accuracy, revision potential, and control variables. In Model 2, feedback features were added on the basis of Model 1. In Model 3, feedback focus was added on the basis of Model 1. Model 4 was the full model examining the effects of all the variables on implementation, and it provided a better fit than the previous three models: χ^2^ (11) = 850.0, *p* < 0.001.

**Table 6:** Logistic regression analysis of the effect of the two dimensions of feedback quality (accuracy and revision potential), features, and focus on implementation.

Variable	Two dimensions of feedback quality (Model 1)			Two dimensions of feedback quality + features (Model 2)			Two dimensions of feedback quality + focus (Model 3)			Two dimensions of feedback quality + features + focus (Model 4)		
	*B*	*SE*	*OR*	*B*	*SE*	*OR*	*B*	*SE*	*OR*	*B*	*SE*	*OR*
Accuracy	0.72	0.03	2.05***	0.77	0.04	2.17***	0.64	0.03	1.89***	0.78	0.04	2.18***
Revision potential	0.02	0.04	1.02	0.16	0.05	1.17***	0.26	0.05	1.13***	0.28	0.05	1.32***
Feedback features												
Identification	–	–	–	0.19	0.09	1.20*	–	–	–	0.22	0.09	1.24*
Suggestion	–	–	–	−0.99	0.10	0.37***	–	–	–	−0.96	0.10	0.38***
Solution	–	–	–	0.10	0.11	1.10	–	–	–	−0.13	0.12	0.88
Explanation	–	–	–	−0.17	0.18	0.84	–	–	–	−0.17	0.18	0.85
Mitigating praise	–	–	–	0.06	0.30	1.06	–	–	–	0.10	0.30	1.11
Feedback focus												
Meaning-level (reference: surface-level)	–	–	–	–	–	–	−0.75	0.07	0.47***	−0.63	0.08	0.53***
Control variables												
Gender	0.12	0.11	1.13	0.11	0.11	1.12	0.13	0.11	1.13	0.12	0.11	1.12
Comment length	−0.01	0.00	0.99***	−0.01	0.00	0.99***	−0.01	0.00	0.99**	−0.01	0.00	0.99*
Draft 1 quality	0.00	0.01	1.00	0.00	0.01	1.00	0.00	0.01	1.00	0.00	0.01	1.00
Model fit statistics												
AIC	6789.20			6571.34			6681.57			6509.94		
BIC	6835.62			6650.92			6734.62			6595.15		

*N* = 5,606. “–” means that the variable was not included in the model.

*p < 0.05, **p < 0.01, ***p < 0.001.

The effect of accuracy was significant and constant across models. Students tended to implement more feedback when it accurately addressed the text problem. The effect of accuracy increased when feedback features were added in Model 2 (*B* = 0.77, *SE* = 0.04, *p* < 0.001), and the effect was weakest when only feedback focus was included in Model 3 (*B* = 0.64, *SE* = 0.03, *p* < 0.001). In the full model, among all the factors predicting feedback implementation, the effect of accuracy was the largest among all the variables (*B* = 0.78, *SE* = 0.04, *p* < 0.001). The *OR* value of accuracy reached 2.18 in Model 4, suggesting that feedback with an extra point in accuracy was 2.18 times more likely to be implemented than feedback with a point less. Revision potential was not significant in Model 1 (*B* = 0.02, *SE* = 0.04, *p >* 0.05), but its effect became significant when feedback features and focus were included in Models 2–4. This indicated that revision potential did not predict feedback implementation together with the control variables, but when the effects of feedback features and focus were taken into consideration, revision potential became a significant predictor.

In the full model, among feedback features, identification positively contributed to feedback implementation (*B* = 0.22, *SE* = 0.09, *p* < 0.05). Suggestion was negatively significant (*B* = −0.96, *SE* = 0.10, *p* < 0.001). Surprisingly, the effects of solution, explanation, and mitigating praise were not significant in either of the two models that involved feedback features (Model 2 and Model 4).

Compared with surface-level peer feedback, meaning-level peer feedback significantly led to less implementation in this study (Model 3 and Model 4). Among the control variables, only comment length negatively predicted implementation (*B* = −0.01, *SE* = 0.00, *p* < 0.01). Gender and first draft quality were not significant predictors.

### Relationship between the two-dimensional feedback quality and implementation

In general, average peer feedback quality (*M* = 3.36, *SD* = 1.93) was unsatisfactorily at a low level (getting about 56% of the total rating), with a big variation between high and low quality feedback (*Max* = 4.32, *Min* = 2.36). Of a total of 6 points, 28.5% of feedback (*N* = 1,600) was at the assigned 6 or 5 points range, 43.2% (*N* = 2,421) got 4 or 3 points, and 28.3% (*N* = 1,585) got 2 points or less. This indicated that nearly 30% of feedback was very poor in quality which was either not aligned with/incorrectly addressed text problems or had low potential for writing improvement, or both.

**Figure 1:**
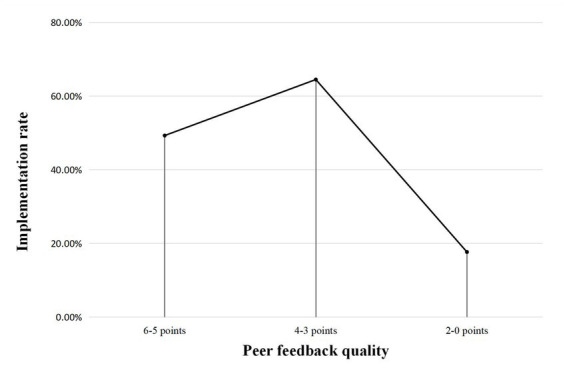
Mean feedback implementation rate of high, middle-range, and low-quality feedback (*N* = 5,606).

Different from common expectations and previous research findings (Wu and Schunn, 2020a), implementation rates were found to be highest (over 60%) for middle-range quality feedback (4–3 points) and lowest (17.7%) for low-quality feedback (2–0 points) in this study (Figure 1). Apparently, the students were able to screen out and discard most of the low-quality feedback in their text revision. However, they also ignored a large proportion (52.3%) of high-quality feedback (accurate feedback with high revision potential). Ideally, high-quality feedback deals with more complex issues of writing and therefore is more helpful to writing improvement if implemented.

In order to identify potential confounds and multicollinearity problems among the variables, Pearson correlation analysis was carried out (Table 7). Peer feedback quality was significantly related to feedback implementation (*r* = 0.35**).

**Table 7:** Correlations among peer feedback quality, features, focus, and implementation.

	Variable	1	2	3	4	5	6	7	8	9	10
1	Feedback quality										
2	Identification	−0.02									
3	Suggestion	0.30**	−0.21**								
4	Solution	−0.14**	−0.45**	−0.49**							
5	Explanation	0.07**	−0.04**	−0.01	−0.01						
6	Mitigating praise	0.03**	−0.02	0.07**	−0.06**	0.01					
7	Meaning-level (reference: surface-level)	0.22**	0.29**	0.32**	−0.56**	0.04**	0.07**				
8	Gender	−0.02	0.02	−0.01	0.01	−0.03**	−0.02	−0.02			
9	Comment length	0.08**	0.08**	0.13**	0.07**	0.22**	0.09**	0.15**	−0.03*		
10	Draft 1 quality	−0.07**	−0.02	0.02	0.01	−0.01	−0.02	−0.02*	0.20**	−0.04**	
11	Implementation	0.35**	−0.01	−0.08**	0.11**	0.01	−0.01	−0.11**	0.02	−0.01	−0.02*

*p < 0.05, **p < 0.01.

To answer the second research question, we conducted the second set of logistic regressions (Table 8). Model 5 tested the relationship between feedback quality and implementation together with control variables. Model 6 and Model 7 tested the relationship when feedback features alone or focus alone was included. Model 8 included all the variables and provided a better fit to the data: χ^2^(10) = 812.11, *p* < 0.001.

**Table 8:** Logistic regression analysis of the effect of peer feedback quality, features, and focus on implementation.

Variable	Feedback quality (Model 5)			Feedback quality + features (Model 6)			Feedback quality + focus (Model 7)			Feedback quality + features + focus (Model 8)		
	*B*	*SE*	*OR*	*B*	*SE*	*OR*	*B*	*SE*	*OR*	*B*	*SE*	*OR*
Feedback quality	0.42	0.02	1.52***	0.54	0.02	1.71***	0.49	0.02	1.64***	0.58	0.02	1.78***
Feedback features												
Identification	–	–	–	0.18	0.08	1.20*	–	–	–	0.22	0.09	1.25*
Suggestion	–	–	–	−0.80	0.10	0.45***	–	–	–	−0.82	0.10	0.44***
Solution	–	–	–	0.57	0.10	1.77***	–	–	–	0.17	0.11	1.18
Explanation	–	–	–	−0.07	0.18	0.93	–	–	–	−0.09	0.18	0.92
Mitigating praise	–	–	–	−0.04	0.30	0.96	–	–	–	0.05	0.30	1.05
Feedback focus												
Meaning-level (reference: surface-level)	–	–	–	–	–	–	−0.95	0.06	0.39***	−0.76	0.08	0.47***
Control variables												
Gender	0.15	0.11	1.16	0.13	0.11	1.14	0.14	0.11	1.15	0.13	0.11	1.14
Comment length	−0.01	0.00	0.99***	−0.01	0.00	0.99***	−0.01	0.00	0.99**	−0.01	0.00	0.99*
Draft 1 quality	0.00	0.01	1.00	0.00	0.01	1.00	0.00	0.01	1.00	0.00	0.01	1.00
Model fit statistics												
AIC	6935.63			6649.04			6712.02			6551.55		
BIC	6975.42			6721.99			6758.44			6631.12		

*N* = 5,606. “–” means that the variables were not included in the model.

*p < 0.05, **p < 0.01, ***p < 0.001.

In Model 5, feedback quality significantly predicted implementation (*B* = 0.42, *SE* = 0.02, *p* < 0.001). When feedback quality increased by one point, the feedback was 1.52 times (*OR* = 1.52) as likely to be implemented than feedback with one point less. Adding feedback features or focus to the models (Model 6 and 7) did not change the estimated relationships between feedback quality and implementation. When feedback quality, features, and focus were all included (Model 8), feedback quality remained to be a significant predictor with the largest effect (*B* = 0.58, *SE* = 0.02, *p* < 0.001). Specifically, when feedback quality increased by one point, it was 1.78 times (*OR* = 1.78) as likely to be implemented than feedback quality with one point less (Model 8).

In terms of feedback features, identification positively predicted feedback implementation in the full model (*B* = 0.22, *SE* = 0.09, *p* < 0.05). On the contrary, there was a negative relationship between suggestion and feedback implementation (Model 6 and Model 8). Solution, explanation and mitigating praise were not significantly related to implementation in the full model. Similar to that in the first set of regression (Model 3 and Model 4), surface-level peer feedback more significantly predicted implementation (Model 7 and Model 8) and among the control variables, only comment length negatively predicted implementation.

## Discussion

In line with Wu and Schunn’s (2020a) study, the current study also deems that the collaborative peer review activities benefit learning in nature (Hovardas et al., 2014; Wu and Schunn, 2020a). The collaboration in peer review acts as a social process in which students work together to handle a writing task that no single hand could reach the intended achievement. The developmental changes experienced by individual ESL learners first occur between peers and then internally within the individual. To better understand and improve the effectiveness of this interactive peer feedback process, this study further explored the issue of peer feedback quality and its impact on feedback implementation.

As the purpose of peer review is to improve writing by involving students actively in providing and receiving feedback, we argue that peer feedback quality should be measured in terms of its degree of helpfulness for text improvement. Inspired by previous studies (van Steendam et al., 2010; Gao et al., 2019; Wu and Schunn, 2020a), the current study ventured further to explicate the nature of peer feedback quality by examining quantitatively what instructors and students care most in peer review: the accuracy (both feedback alignment with original text problems and correct addressing of text problem) and helpfulness (the potential of leading to meaningful revision) of peer feedback for writing improvement. Informed particularly by Wu and Schunn’s (2020a) study, this conceptualization of feedback quality highlights the potential function of peer feedback in facilitating revision and writing improvement. The combination of feedback accuracy and revision potential may be closest to the sense of effectiveness that teachers and students value most as a measure of peer feedback’s effectiveness. Different from using an overall judgment as in Wu and Schunn’s (2020a) study, the designed measurement scale in the current study provides a more detailed measurement and specifies the process of evaluating feedback quality using a four-level rating scale (0–3) for each dimension of peer feedback quality. Practically, the measurement scale serves as a useful tool for teachers when assessing students’ feedback quality.

The overall low peer feedback quality (medium in accuracy and low in revision potential) indicated that peer feedback was sometimes not satisfactory (Carson and Nelson, 1996; Tsui and Ng, 2000; Walvoord et al., 2007; Misiejuk and Wasson, 2021). Similarly, Hovardas et al. (2014) also found that the majority of peer feedback were scientifically accurate, but insufficient with suggestions and explanations for changes and improvement of writing skills. Therefore, although feedback accuracy was of medium level, the low level of revision potential suggested that students tended to receive feedback with the potential of leading to only minor writing improvement (Allen and Mills, 2014; Gao et al., 2019). Additionally, the inconsistency between accuracy and revision potential suggested that accuracy or the revision potential alone may not fully reflect the helpfulness of feedback on revision improvement. Accurate feedback with limited revision potential may have limited strength to improve revision quality and vice versa. Therefore, measuring feedback quality using either one of these two dimensions only reveals one side of the coin, which further suggests that the proposed two-dimensional measurement scale is a valid means of describing and reporting feedback quality, at least in the EFL context of the current research.

The predictive strength of peer feedback quality on implementation reveals two significant findings. First and foremost, when examining the predictive power of accuracy and revision potential, the largest *OR* values of accuracy (Model 1–4) suggested that feedback accuracy was the central predictor of feedback implementation and hence it should be of priority (Hovardas et al., 2014). The results were consistent with other research (Hovardas et al., 2014; Gao et al., 2019) in which students’ revisions were influenced, either fully or partly, by peer feedback accuracy. Allen and Mills (2014) reported that, although the number of inaccurate feedback was minimal in number, and that only less than half of the erroneous comments were used in revision, the inaccurate feedback negatively affected writing quality. In addition, the large predictive power of feedback accuracy shows that students are highly sensitive to the alignment and the accuracy of the suggested solutions, which should reassure teachers who are hesitant to use peer feedback (Wu and Schunn, 2020a).

Moreover, the inclusion of feedback features and focus did not change the existing relationship between feedback accuracy and feedback implementation, indicating that the effect of feedback accuracy was constant and robust. Revision potential significantly predicted implementation only when feedback features/focus were considered (Model 2–4) and its effect became largest when features and focus were both included (Model 4). Obviously, the inclusion of feedback features and focus changed the observed relationship between revision potential and feedback implementation. The significant relationships among the revision potential, feedback features, and feedback focus indicated that feedback features and focus were crucial statistic confounds that should be considered when exploring the influencing factors of feedback implementation (see Table 5). In Model 1, the omission of feedback features and focus inevitably increased the variance of the error term. After feedback features and focus were included in Models 2–4, the variance of the error term became smaller, and it was probably why revision potential became a significant predictor of feedback implementation (see Table 6). Given the positive effect of accuracy and revision potential on feedback implementation, EFL students should be instructed on detecting the flaws central to text improvement and addressing the flaws in the correct and substantial ways.

Secondly, the largest *OR* values of feedback quality indicated that its effect on implementation was significant and constant across all models (Model 5–8). A crucial message for practice is that, in addition to validity and reliability, which have been the focus of many earlier studies (Falchikov and Goldfinch, 2000; Cho et al., 2006), the quality of feedback can affect its effectiveness (Gielen et al., 2010). The central role of feedback quality in students’ likelihood of feedback implementation is similar to that in Wu and Schunn’s (2020a) study and it also confirms the significance of feedback quality in peer review (Hattie and Timperley, 2007; Walker, 2015). The effect of feedback quality became largest when feedback features and focus were considered together (Model 8). The consideration of feedback features and focus has provided a better explanation of feedback implementation because the reduced AIC-adjusted deviance in the full model (Model 8) in comparison with the baseline model (Model 5) suggested that the full model had stronger explanatory strength to feedback implementation. Compared with previous studies focusing on one or two comment-level factors (e.g., Lu and Law, 2012; Patchan et al., 2016), this study ventures further to explore the effect of multiple factors and their specific contributions to feedback implementation.

Although feedback quality significantly predicted implementation, it was important to note that students tended to implement more middle-range quality feedback. High-quality feedback is undoubtedly more facilitating to revision improvement, but implementing high-quality feedback is more challenging and students might have limited knowledge about how to handle the information delivered through such feedback (Wichmann et al., 2018). Since students also tend to screen out low-quality feedback by employing some decision-making strategies (Gielen et al., 2010; Hovardas et al., 2014), they tend to implement only those middle-range quality feedback which is presumably within their zone of competence. This indicates that more guidance is needed to encourage students to take the tougher task of incorporating high-quality feedback in future instructions.

Although feedback features, focus and other control variables are not the central foci of the current study, we have discussed these variables because they are theoretically and empirically important (Nelson and Schunn, 2009; Allen and Mills, 2014; Patchan et al., 2016). The positive role of identification on implementation in this study was similar to previous studies (Lu and Law, 2012; Wu and Schunn, 2020a). In terms of cognitive load, identifying a text problem is relatively easier than giving a suggestion, a solution, or an explanation, which partially explains why the amount of feedback with identification was the largest in the data. Suggestion was a significant negative predictor of implementation in this study as feedback with suggestion was usually general and sometimes vague for students to comprehend and take action. A follow-up analysis revealed that general advice was not helpful for students to address the text problems. For example, feedback like “*You should change some examples.*” usually ended up being ignored in text revision. Different from the findings in previous studies (Gielen et al., 2010; Wu and Schunn, 2020a), explanation had no effect on feedback implementation in the present study. It was possible that the small amount of explanatory feedback could hardly generate statistical power on feedback implementation. This might also explain why mitigating praise was not a significant predictor. Solution significantly predicted implementation in Model 6, but when feedback focus was jointly considered (Model 8), it turned insignificant, suggesting that feedback focus could mediate the relationship between feedback features and implementation.

Since meaning-level feedback significantly led to less implementation than low-level feedback did, it was obvious that students trended toward taking less challenging tasks (Gao et al., 2019). Students implemented more low-level feedback as meaning-level issues were found to be more difficult for students to address (Ene and Upton, 2014; Patchan et al., 2016). The negative correlation between comment length and implementation again proved that students tended to avoid repairs mentioned in long comments which might involve more suggestions or explanation to solve harder text problems. The findings about feedback features and focus indicate that teachers’ guidance should be directed toward emphasizing the significance of helpful features (e.g., identification of problems) and instructing students to implement more meaning-level feedback.

## Conclusion

This study reveals that peer feedback quality can be more comprehensively and scalably explicated from two dimensions: accuracy and revision potential of feedback. The complexity of the predictive strength of feedback quality on implementation well demonstrates the different and interactive power of peer feedback quality, features, focus, and other variables in peer review. Yet, among all these elements, feedback quality plays a central role in determining peer feedback implementation.

Pedagogically, this study implies that improving peer feedback quality should strategically orient toward both accuracy and revision potential of feedback as accurate feedback with strong revision potential is most likely to lead to revision improvement when implemented. At the same time, while peer feedback training should prioritize feedback quality, special care should be given to encouraging students to take the pain of dealing with complex issues in revision by implementing high-quality feedback, as well as feedback with significant features such as identification of the problem and content/high-level focus feedback. Therefore, to improve the effectiveness of peer review, more importance should be attached to promoting peer feedback literacy in both providing and implementing high-quality feedback.

Some limitations of the present study should be considered. Firstly, with the support of *Peerceptiv,* peer review of this study was conducted anonymously online with participants from one course. As such, the generalization of the results of this study to other contexts involving different participants from other disciplines with different writing tasks should be exercised with caution. Secondly, this study focused on the effects of feedback quality on implementation, leaving the effects of providing high or low-quality feedback on students’ own draft revision unexplored. Future research can adopt this two-dimensional feedback quality measurement to further test the effect of feedback quality on the feedback providers’ learning performance to obtain a more comprehensive understanding of the significance of peer feedback quality in determining the effectiveness of peer review. Lastly, although carefully designed, this study is correlational in nature. In promoting feedback quality, intervention studies are needed in the future, and results from the present study can help locate the intervention foci.

## Data availability statement

The original contributions presented in the study are included in the article/Supplementary material, further inquiries can be directed to the corresponding author.

## Author contributions

WH: conceptualization, methodology, software, data coding and curation, writing—original draft preparation, and writing—reviewing and editing. YG: conceptualization, methodology, writing—reviewing and editing, supervision, and funding acquisition. All authors contributed to the article and approved the submitted version.

## Funding

This work was supported by the Major Program of National Fund of Philosophy and Social Science of China (CN) [grant number 18BYY114].

## Conflict of interest

The authors declare that the research was conducted in the absence of any commercial or financial relationships that could be construed as a potential conflict of interest.

## Publisher’s note

All claims expressed in this article are solely those of the authors and do not necessarily represent those of their affiliated organizations, or those of the publisher, the editors and the reviewers. Any product that may be evaluated in this article, or claim that may be made by its manufacturer, is not guaranteed or endorsed by the publisher.

## Footnotes

^1^ https://peerceptiv.com
