# Supplementary material for: Explicating peer feedback quality and its impact on feedback implementation in EFL writing
Source: Front Psychol. 2023 Jul 14;14:1177094. doi: 10.3389/fpsyg.2023.1177094 (PMC10382203; doi:10.3389/fpsyg.2023.1177094)
Supplement: Supplementary file 1 [file Data_Sheet_1.doc]

**Appendix A Peer review rubrics**

**Dimension 1: Thesis Statement**

*Comments should consider the following aspects:*

1. Is there a specific and clear thesis statement in the introductory paragraph? If you can’t find a thesis statement, can you summarize what you have read so far and show your partner how to improve the thesis statement?
2. How does the thesis statement align with the following paragraphs? Do you think the thesis statement can summarize the main ideas of the body paragraphs?
3. Does the writer provide background information in the first paragraph?

**Dimension 2: Organization**

*Comments should consider the following aspects:*

1. How well is the paper developed around a main idea? Is there a paragraph that is not connected to the main point?
2. Should the three body paragraphs be re-ordered? Why or why not?
3. Does each body paragraph state a topic sentence and develop it? If you have found the absence of topic sentence in some paragraphs, make suggestions for your partner to add it.
4. How strongly/weakly do transitions connect the body paragraphs? What does the writer need to do to improve the transitions of the passage?
5. What and where should linking words be added or changed to improve the transitions between all sentences in a paragraph?

**Dimension 3: Argument**

*Comments should consider the following aspects:*

1. How well does the writer use details to illustrate ideas in the body paragraphs? Do you think the details are accurate, complete and sufficient? If not, please give your advice.
2. Do you think the writer make full explanation of the examples? Do you think his explanation support the topic sentence?
3. Does the writer mention any counter arguments about the discussed issues? If so, identify some. If not, give a suggestion on how to improve argument components.
4. How does the writer restate the major points (but different wording) at the end of the passage? Does the conclusion contain too much irrelevant information to the thesis statement? If yes, make a suggestion.

**Dimension 4: Grammar and Vocabulary**

*Comments should consider the following aspects:*

1. To what extent does content of the essay effectively address the writing task? You can comment on the following aspects: the number of words, the title, the number of paragraphs, etc.
2. Does the essay conform to the writing conventions in terms of punctuation, capitalization, abbreviations, etc.?
3. Can you identify some long sentences that you can not understand and try to make some suggestions for the writer to rewrite them?
4. Is there a fragment that lacks a subject or a verb and fails to express a complete thought? Make some suggestions.
5. Can you identify some errors and provide some suggestions on the use of words?
6. Can you give some comments on how does the writer use different kinds of sentences such as simple sentence, complex sentence, attributive clause, etc? If the writer just use one kind of sentence, how can he/she improve? Can you give the writer some examples?
