# Supplementary material for: Explicating peer feedback quality and its impact on feedback implementation in EFL writing
Source: Front Psychol. 2023 Jul 14;14:1177094. doi: 10.3389/fpsyg.2023.1177094 (PMC10382203; doi:10.3389/fpsyg.2023.1177094)
Supplement: Supplementary file 2 [file Table_1.doc]

**Appendix B Training steps**

| **Goal** | **Training activity** | **Duration** |
| --- | --- | --- |
| To guide students to register on *Peerceptiv* and to join the class set up by the researcher on *Peerceptiv* | Watching a short video introducing *Peerceptiv* | 15 min |
| To show students what to comment on and how to provide peer feedback effectively | Teacher modeling (introducing commenting strategies with the help of the review rubrics; analyzing sample essays and components of high-quality essays and feedback) | 60 min |
| To guide students to improve feedback quality | Teacher lectures (introducing the benefits and ways of being a good reviewer; illustrating the principles of reviewing with good examples and bad examples) | 30 min |
| To guide students to implement high-quality feedback | Teacher-guided discussion on implementing feedback to improve writing | 30 min |
